# Sustainability Assessment of Green Ammonia Production To Promote Industrial Decarbonization in Spain

**DOI:** 10.1021/acssuschemeng.3c04694

**Published:** 2023-10-25

**Authors:** Sergi Vinardell, Palina Nicolas, Ana María Sastre, Jose Luis Cortina, César Valderrama

**Affiliations:** † Chemical Engineering Department, Escola d’Enginyeria de Barcelona Est (EEBE), Universitat Politècnica de Catalunya (UPC)-BarcelonaTECH, C/Eduard Maristany 10-14, Campus Diagonal-Besòs, 08930 Barcelona, Spain; ‡ Barcelona Research Center for Multiscale Science and Engineering, Campus Diagonal-Besòs, 08930 Barcelona, Spain; § CETaqua, Carretera d’Esplugues, 75, 08940 Cornellà de Llobregat, Spain

**Keywords:** green ammonia, green hydrogen, life cycle assessment (LCA), techno-economic evaluation, water-energy nexus, sustainable resource management, renewable energy

## Abstract

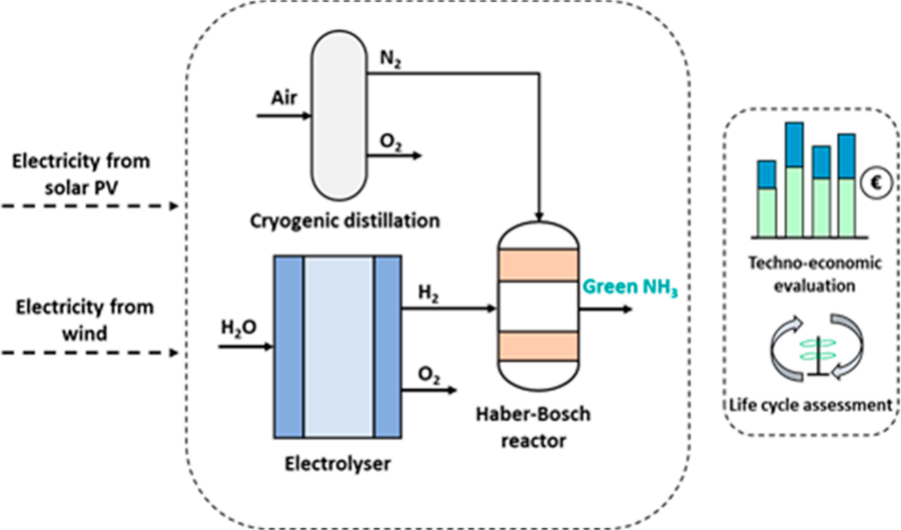

This article investigates the economic and environmental implications of implementing green ammonia production plants in Spain. To this end, one business-as-usual scenario for gray ammonia production was compared with three green ammonia scenarios powered with different renewable energy sources (i.e., solar photovoltaic (PV), wind, and a combination of solar PV and wind). The results illustrated that green ammonia scenarios reduced the environmental impacts in global warming, stratospheric ozone depletion, and fossil resource scarcity when compared with conventional gray ammonia scenario. Conversely, green ammonia implementation increased the environmental impacts in the categories of land use, mineral resource scarcity, freshwater eutrophication, and terrestrial acidification. The techno-economic analysis revealed that the conventional gray ammonia scenario featured lower costs than green ammonia scenarios when considering a moderate natural gas cost. However, green ammonia implementation became the most economically favorable option when the natural gas cost and carbon prices increased. Finally, the results showed that developing efficient ammonia-fueled systems is important to make green ammonia a relevant energy vector when considering the entire supply chain (production/transportation). Overall, the results of this research demonstrate that green ammonia could play an important role in future decarbonization scenarios.

## Abstract 2

Green ammonia production using wind and solar renewable energy is important to improve the sustainability of the ammonia industry.

## Introduction

Industrial decarbonization is a priority for European Union (EU) policy-makers. The European Green Deal has established the ambitious objectives of (i) reducing by 55% the net greenhouse gas emissions by 2030 and (ii) achieving net-zero greenhouse gas emissions by 2050.^1^ However, these objectives are still far from being accomplished since many industrial sectors are energy-intensive and have high emissions of greenhouse gases. In this regard, the Haber–Bosch (HB) process is a critical process contributor because it consumes 1–2% of the world’s energy and represents 1% of carbon emissions.^2^ The conventional HB has been used since the early 20th century to produce NH_3_ from hydrogen (H_2_) and atmospheric nitrogen (N_2_). This process represented a revolution in the production of synthetic fertilizers since its implementation faced food insecurity and exponential population growth.^3^ However, considering the current climate emergency situation, it is crucial to look for technological solutions to update the conventional HB process to a more sustainable solution for ammonia production.

The technology used to produce H_2_ is the most critical factor determining the sustainability of conventional ammonia production. In the HB process, steam methane reforming (SMR) is the best available technique to produce the H_2_ needed for the subsequent HB reaction.^4^ To date, 76% of the H_2_ needed for ammonia manufacturing is produced using the SMR process.^3^ This SMR process consists of the reaction of CH_4_ with steam under highly endothermic conditions. However, the conventional HB process using the SMR method does not fit with the current decarbonization strategy established by the EU since (i) it produces H_2_ using nonrenewable natural gas, (ii) it is a highly energy-intensive process, and (iii) it leads to high emissions of greenhouse gases up to 2.5 t_CO2_-_eq_/t_NH3_.^4,5^

To address the challenges associated with the conventional SMR method, the HB process is being reconsidered to decrease its carbon footprint and the reliance on carbon-based fuels. In this regard, the production of green ammonia has emerged as one of the most relevant topics to achieve carbon neutrality for ammonia production.^6,7^ Unlike gray and blue ammonia, which use the conventional SMR process for H_2_ production, green ammonia is obtained using the green H_2_ produced during water electrolysis powered with renewable electricity.^8^ Nitrogen is obtained after its separation from air using pressure swing adsorption (PSA) or cryogenic distillation.^9^ Ammonia production from alternative H_2_-producing methods has the potential to reduce greenhouse gas emissions up to 70% in comparison with the conventional SMR process.^10^ However, the green ammonia production process is still at an earlier stage of development when compared with the highly mature gray ammonia production process (Technology Readiness Level (TRL) of 9).^11^ For this reason, holistic studies are necessary to understand whether green ammonia implementation could become a more sustainable alternative for ammonia production considering both economic and environmental factors.

Different studies have evaluated the environmental and economic effects of producing green ammonia using renewable energy. Zhang et al.^12^ conducted a techno-economic evaluation of two different alternatives for green ammonia production. Bicer et al.^10^ compared the impact of four different energy sources on the environmental prospect of producing ammonia via water electrolysis and the HB process. Egerer et al.^13^ evaluated the economics of using green ammonia as a potential energy vector to promote decarbonization. However, limited information is available in the literature concerning the importance of geographical location, renewable energy source, natural gas cost, or carbon pricing on the environmental and economic feasibility of implementing green ammonia projects when compared with gray ammonia projects.

The present study aims to evaluate the environmental and economic implications of implementing green ammonia production using renewable energy sources. To this end, life cycle assessment (LCA) and techno-economic assessment (TEA) were conducted from a theoretical green ammonia production plant implemented in Spain. The present paper expands the current knowledge on green ammonia since it evaluates: (i) the technical, environmental, and economic feasibility of green ammonia implementation in Spain; (ii) the role of the renewable energy source in the environmental and economic prospects of green ammonia application; (iii) the impact of natural gas cost and carbon pricing on the ammonia production costs; and (iv) the economic implications of using green ammonia as an energy vector in Europe.

## Materials and Methods

### Case Study Description

This theoretical study compares the economic and environmental performances of gray and green ammonia production plants implemented in Spain. The plants were designed to produce 430 kt of ammonia per year.^14^ This size was selected because it represents a high-sized green ammonia production plant and can be representative for future implementation of green ammonia projects. The plant was considered to operate 8000 h/year with an utilization factor of 0.92.^15^ The schematic representation of gray and green ammonia production plants can be found in Figure S1 of the Supporting Information. The description of the different scenarios is summarized as follows.

#### Baseline Scenario

The Baseline Scenario represents a standard gray ammonia thermally driven production plant using SMR for hydrogen production. The SMR process takes place in two SMR reactors: (i) the first SMR reactor, which is operated at 850–900 °C and 25–35 bar under allothermal conditions, and (ii) the second SMR reactor, which is operated at 900–1000 °C under autothermal conditions.^4^ The energy needed to operate the process is obtained from the external combustion of natural gas. In the second SMR reactor, air is compressed and introduced to achieve the partial oxidation conditions needed to maintain the autothermal conditions and to provide the nitrogen needed for the HB reaction. Besides H_2_ and N_2_, other gases such as carbon monoxide (CO) or unreacted methane are present in the outlet gas mixture. For this reason, a multistep process before the HB process is needed to maximize the conversion to H_2_ and to minimize the impurities of the gas mixture (e.g., CO, CO_2_, H_2_O) that could lead to catalyst poisoning in the ammonia synthesis reactor.^9^ This multistep process consists of three different systems: (i) water–gas shift system, in which the unreacted carbon monoxide, methane, and steam are converted into hydrogen; (ii) CO_2_ scrubber, to remove the CO_2_ from the gas mixture; and (iii) methanation, to convert the remaining trace amounts of CO and CO_2_ into methane.^4,16^ Subsequently, the ammonia is produced in the HB process through the reaction of the H_2_ and N_2_ under high temperature (450–600 °C) and pressure (100–250 bar) conditions and with the use of an iron-based catalyst.^17^ The energy required to keep the temperature and pressure conditions in the HB process is obtained from the waste heat from the SMR process,^4^ which allows reducing and optimizing the thermal external energy required for gray ammonia production. The high temperature required to increase the kinetics of the HB reaction reduces the conversion of hydrogen according to Le Chatelier principle.^16^ For this reason, a recycle loop is introduced to increase the conversion yield after separating the ammonia produced by means of condensation.^4^ Further information on the scheme for conventional gray ammonia production can be found elsewhere.^4,16^ The conventional HB process is highly dependent on the natural gas price since 4.9 N m^3^ of natural gas is needed to produce 1 kg of H_2_ in the SMR process.^15^ Current natural gas prices in Europe are highly volatile, as a result of geopolitical situations and conflicts. For this reason, two alternatives were included in the Baseline Scenario to evaluate the influence of the natural gas price on the gray ammonia production cost: (i) Alternative A, in which the average 2021 natural gas cost in Spain (28.8 €/MWh) was considered (low-cost alternative), and (ii) Alternative B, in which the average 2022 natural gas cost in Spain (88.9 €/MWh) was considered (high-cost alternative).^18^

#### Scenarios 1, 2, and 3

Scenarios 1, 2, and 3 represent the green ammonia production plant using a water electrolysis process for hydrogen production and powered with renewable electricity. The water electrolysis process was considered to take place in a polymer electrolyte membrane (PEM) electrolyzer.^15^ This typology of electrolyzer was selected since it can be operated under fluctuating energy supply from renewable energy sources.^19,20^ The hydrogen produced through electrolysis was stored in a hydrogen storage system.^14^ Cryogenic distillation was used to separate the nitrogen from the air before the HB process.^9^ Besides nitrogen and hydrogen, oxygen is also produced in both electrolyzer and cryogenic distillation processes. Subsequently, the hydrogen and nitrogen are fed into the HB loop powered with renewable electricity.^4^ In 2030, it is expected that wind and solar PV will be the prevalent sources of electricity generation in Spain.^21^ In this study, three potential off-grid scenarios were considered depending on the renewable energy source: (i) Scenario 1, in which 100% of the electricity comes from wind turbines; (ii) Scenario 2, in which 100% of the electricity comes from solar PV; and (iii) Scenario 3, in which the electricity comes from both wind turbines and solar PV (50:50%). Lithium-ion (Li-ion) batteries were used to ensure a continuous supply of electricity in the green ammonia production system, and its capacity was estimated considering the expected annual operating hours of wind onshore (2300 h) and PV (1800 h) systems in Spain.^21^

### Life Cycle Analysis

The LCA was carried out according to UNE-EN ISO 14040 and 14044.^22,23^ In this section, the different phases of the LCA are described.

#### Goal and Scope Definition

The main goal of this LCA is to evaluate the environmental implications of producing green ammonia from renewable energy sources when compared with conventional gray ammonia production. For this reason, the functional unit (FU) for this study was 1 kg of ammonia produced.

**Figure 1:**
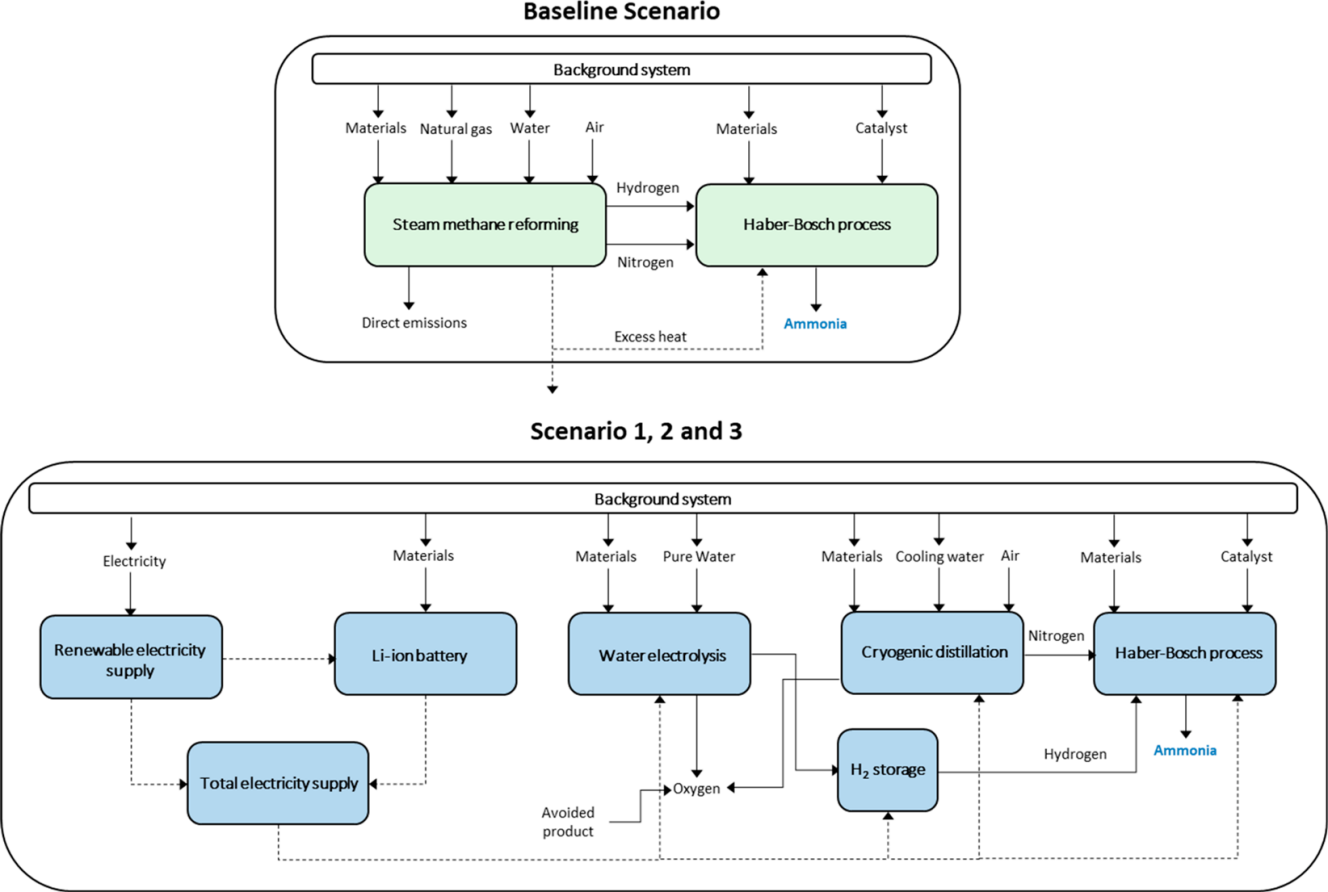
System boundaries of Baseline Scenario (top) and Scenarios 1, 2, and 3 (bottom).

The system boundaries of this study are depicted in Figure 1. The system boundaries include the construction and operation phases of gray and green ammonia production scenarios. The background processes needed for natural gas, water, iron-based catalyst, electricity and materials for construction were included. The scope of the analysis is defined as cradle-to-gate since the end-use impacts regarding the final application of the produced ammonia were not included. Besides ammonia, oxygen byproduct is also produced in the electrolyzer and cryogenic distillation stages. To address the multifunctionality issue, a system expansion was used in which the avoided burdens from the oxygen produced are included.^15,24^ It was assumed that the excess heat from the SMR process in the Baseline Scenario covered the energy requirements of the HB process.^4^ It is worth mentioning that the SMR process in Figure 1 includes the SMR reactors, water–gas shift, CO_2_ scrubber, and methanation stages needed before the HB stage. Detailed description of the four scenarios under evaluation can be found in the Case Study Description section.

#### Life Cycle Inventory

The life cycle inventory data of the different inputs and outputs included in the LCA for the different scenarios can be found in Table S1. The inventory list was calculated from the average literature values. The inventory data from the background processes for all scenarios were obtained from Ecoinvent database v3.7 (see Table S2).

#### Life Cycle Impact Assessment

The characterization and classification stages of the life cycle impact assessment (LCIA) were conducted using the ReCIPe 2016 Midpoint Hierarchist method. The study was focused on seven different impact categories relevant to understanding the environmental impacts of green ammonia production implementation: (i) global warming (kg CO_2eq_); (ii) fossil resource scarcity (kg oil_eq_); (iii) freshwater eutrophication (kg P_eq_); (iv) mineral resource scarcity (kg Cu_eq_); (v) stratospheric ozone depletion (kg CFC11_eq_); (vi) terrestrial acidification (kg SO_2eq_); and (vii) land use (m^2^a crop_eq_).

### Techno-economic Evaluation

The techno-economic evaluation was performed to analyze the economic implications of producing green ammonia under different renewable energy source scenarios when compared with conventional gray ammonia production. The evaluation included the capital expenditures (CAPEX), operating expenditures (OPEX), and revenue for the different scenarios. Regarding the Baseline Scenario, the costs included (i) the CAPEX required to construct the SMR and HB processes and (ii) the OPEX required to purchase the natural gas, water, and iron catalyst, as well as the labor and maintenance derived from the operation of the plant. Regarding Scenarios 1, 2, and 3, the costs included (i) the CAPEX required to construct the electrolyzer, cryogenic distiller, Li-ion battery system, hydrogen storing tanks, and HB process; and (ii) the OPEX required to obtain the electricity from the renewable energy sources, water, and iron catalyst, as well as the labor and maintenance derived from the operation of the plant. The potential revenue obtained from oxygen produced in electrolysis and cryogenic distillation systems was also included in these scenarios. Detailed information about the different cost parameters considered for the evaluation can be found in Table S3. Subsequently, the capital recovery factor was obtained (eq 1) and the net cost of the different scenarios was calculated after subtracting the gross cost (annualized CAPEX and OPEX) from the achieved revenues (eq 2). Finally, the ammonia cost was calculated according to eq 3.
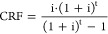
1
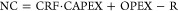
2
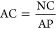
3where CRF is the capital recovery factor (−); NC is the net cost (€/year); CAPEX is the capital expenditure (€); OPEX is the operating expenditure (€/year); R is the oxygen revenue achieved in Scenarios 1, 2, and 3 (€/year); i is the discount rate (%); t is the project lifetime (20 years); AC is the ammonia production cost (€/kg_NH3_); and AP is the annual ammonia production (kg_NH3_/year). Two different discount rates were considered for gray and green ammonia production processes due to the different levels of maturity of both systems. The discount rate of gray ammonia production was assumed to be 5%, while the discount rate of green ammonia production was assumed to be 7% based on average literature values.^7^

### Sensitivity Analysis

A sensitivity analysis was conducted for a ±30% variation of the most important economic and environmental input parameters.^25^ The sensitivity analysis was included to obtain the impact of the input parameters on the ammonia cost and global warming impact category. The results of the sensitivity analysis can be found in Figure S2.

## Results and Discussion

### Life Cycle Assessment

**Figure 2:**
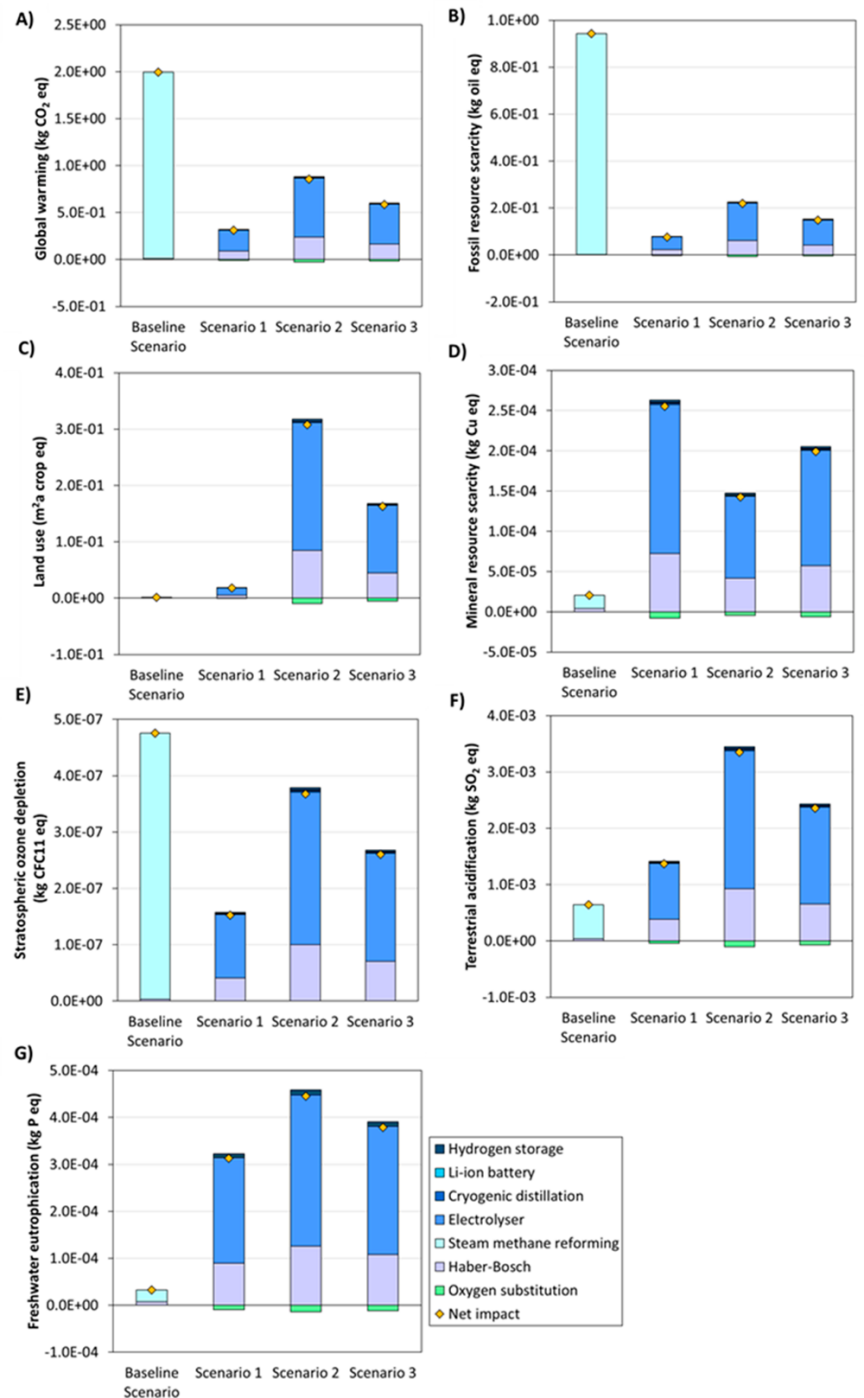
Net environmental impact and contribution of the different processes in (A) global warming, (B) fossil resource scarcity, (C) land use, (D) mineral resource scarcity, (E) stratospheric ozone depletion, (F) terrestrial acidification, and (G) freshwater eutrophication. The results of the Baseline Scenario (gray ammonia scenario) and the three green ammonia scenarios (Scenario 1: 100% wind energy; Scenario 2: 100% solar energy, and Scenario 3: 50% solar and 50% wind energy) are referred to the functional unit (1 kg NH_3_ produced).

Figure 2 shows the environmental results of producing green ammonia for the different impact categories under study. The results showed that Scenarios 1, 2, and 3 (green ammonia) substantially reduced the global warming impact category when compared with the Baseline Scenario (gray ammonia). Green ammonia production does not lead to direct emissions of greenhouse gases since (i) it uses green hydrogen produced from the electrolysis of water rather than from the SMR process and (ii) it is powered by the electricity obtained from renewable sources.^4,9^ These results agree with Bicer et al.^10^ and Lee et al.,^24^ who reported important reductions in greenhouse gas emissions when producing ammonia from renewable energy sources. Similarly, the sensitivity analysis showed that direct emissions were the most sensitive environmental parameter in the global warming impact category for the Baseline Scenario (Figure S2). The Baseline Scenario featured a higher impact in the stratospheric ozone depletion category when compared with Scenarios 1, 2, and 3. This demonstrates that green ammonia production would improve the impact categories related to climate change and air quality.

The application of renewable energy sources for ammonia production also reduced the impact concerning the fossil resource scarcity category. Gray ammonia production requires high amounts of natural gas (i) to produce H_2_ in the SMR reactors (17.7 GJ/t_NH3_) and (ii) to obtain the energy required for the SMR reactors (4.5 GJ/t_NH3_).^4^ From these results, it is conceivable to state that green ammonia implementation has a large potential to reduce the dependency on fossil natural gas when compared with conventional gray ammonia production. Finally, it is worth mentioning that the SMR process was the most important contributor in all of the impact categories of the Baseline Scenario because (i) this process consumes large amounts of natural gas for H_2_ production and (ii) the energy required in the HB loop is obtained from the waste heat of the SMR process.

Scenarios 1, 2, and 3 featured higher environmental impacts than the Baseline Scenario in mineral resource scarcity, land use, terrestrial acidification, and freshwater eutrophication categories (Figure 2). Green ammonia production powered with renewable energy sources increases the consumption of minerals with a direct impact on the mineral resource scarcity category since: (i) PV panels and wind turbines require high amounts of minerals for their construction;^26^ (ii) the water electrolyzer consumes large amounts of electricity (∼ 55 kWh/kg H_2_), which implies that intensive renewable energy supply systems would be needed to meet the energy demand;^15^ and (iii) Li-ion battery systems are needed to deal with the fluctuating energy production from renewable energy systems.^14^ It is worth mentioning that high consumption of minerals has been identified as one of the main economic and environmental challenges to implement renewable energy systems.^27,28^ For this reason, the development of technologies aimed at recovering critical raw materials needed for the energy transition will be paramount for the successful implementation of decarbonized chemical processes, such as green ammonia production. The land use impact category also increased after implementing green ammonia production (Figure 2). The higher impact in the land use category can be attributed to the large land requirements needed to implement the renewable energy production systems.^29^ In addition to the land use category, large surface requirements can increase the CO_2_ emissions when the vegetation is cleared or soils are disturbed after implementing these systems.^30,31^ Finally, it is worth mentioning that the implementation of green ammonia would also increase the environmental impact in the freshwater eutrophication and terrestrial acidification categories. Similar outputs have been obtained in the literature for these categories after implementing renewable energy systems such as solar PV.^32^

Scenario 2 was the most impactful green ammonia alternative in the global warming, fossil resource scarcity, land use, stratospheric ozone depletion, terrestrial acidification, and freshwater eutrophication impact categories (Figure 2). In Scenario 2, the electricity needed to operate the green ammonia production plant is fully obtained from solar PV systems. This suggests that green ammonia production using solar PV systems featured a less favorable environmental prospect when compared with wind systems. These results agree with Asdrubali et al.^32^ and Evans et al.,^33^ who observed that energy production from wind featured a lower environmental impact than energy production from solar PV. However, the implementation of wind renewable energy systems features higher costs than solar PV renewable energy systems for ammonia production in Spain (see next section). This output highlights the importance of finding a compromise solution between environmental impacts and costs when implementing renewable energy systems to power green ammonia production plants. Scenario 1 was the most impactful scenario in the mineral resource scarcity category, which can be attributed to the high amounts of common metals (i.e., iron, aluminum, or cooper) and rare earth elements needed to produce wind energy systems.^26^ Finally, it is worth mentioning that electrolysis followed by the Haber–Bosch process were the most important environmental contributors in all of the impact categories of Scenarios 1, 2, and 3, since these processes consume high amounts of electricity for their operation.

### Techno-economic Evaluation

**Figure 3:**
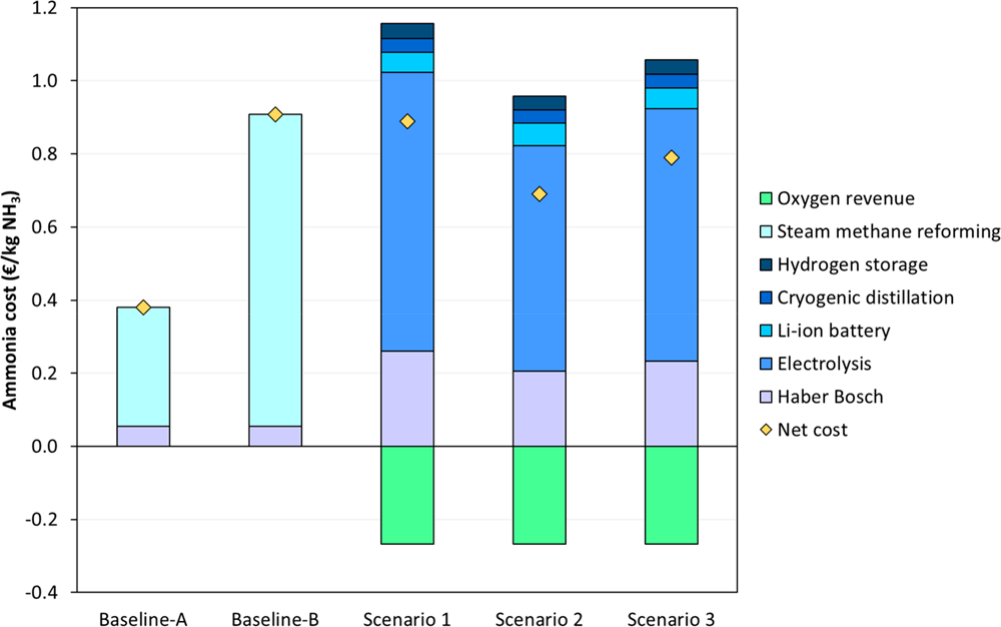
Net cost, gross cost, and revenue of the different processes for the production of 1 kg of ammonia for the two gray ammonia scenarios (Baseline Scenario A, natural gas cost of 28.8 €/MWh; and Baseline Scenario B, natural gas cost of 88.9 €/MWh) and the three green ammonia scenarios (Scenario 1, 100% wind energy; Scenario 2, 100% solar energy; and Scenario 3, 50% solar and 50% wind energy).

Figure 3 illustrates the economic results of gray and green ammonia production scenarios for the different processes. The results show that the Baseline Scenario is the most cost-effective category under a low natural gas cost scenario (Alternative A). These results are in line with Lee et al.^24^ and Nosherwani and Neto,^16^ who also observed higher ammonia production costs in those scenarios powered with renewable energy sources when compared with conventional SMR-HB processes. The high costs of Scenarios 1, 2, and 3 can be primarily attributed to the electrolysis stage, which accounted for more than 60% of the cost in green ammonia scenarios (Figure 3). The high costs of the water electrolysis stage for hydrogen production are mainly caused by (i) the high amount of renewable electricity required to operate the electrolyzer (∼55 kWh/kg H_2_) and (ii) the high capital costs of the PEM electrolyzer (∼1900 €/kW). In this respect, the electricity consumption in the electrolysis process was the most sensitive economic parameter in Scenarios 1, 2, and 3 (Figure S2). The PEM electrolyzer was selected since this hydrogen production system features higher flexibility concerning the load capacity, which is an important feature when considering the fluctuating renewable energy production.^4^ However, the CAPEX of PEM electrolyzers (∼1900 €/kW) is substantially higher than typical alkaline electrolyzers (∼900 €/kW).^19^ Nevertheless, it is worth mentioning that PEM electrolyzers are still at an earlier stage of development (TRL 7–8),^4^ which implies that further improvements in the technology could boost the competitiveness of green ammonia projects.

The net ammonia cost in Scenarios 1, 2, and 3 ranged between 0.6 and 0.9 €/kg_NH3_, which is within the range for different green ammonia production processes.^8,14,24^ Scenario 2 (100% solar energy) was the most cost-effective green ammonia process, followed by Scenario 3 (50% solar and 50% wind energy) and Scenario 1 (100% wind energy). This suggests that using renewable electricity from solar PV energy is the most economically attractive alternative to green ammonia production. Interestingly, the net ammonia cost in green ammonia scenarios was lower than the Baseline Scenario under a high natural gas cost situation (Alternative B). This output is in line with the results of the sensitivity analysis, which illustrated that the natural gas cost featured a relevant impact on the ammonia cost of the Baseline Scenario (Figure S2 of the Supporting Information). These results reinforce the idea that gray ammonia production costs in Spain/EU will be highly uncertain since 83% of the natural gas consumed in EU is imported from other countries^34^ and its cost is highly influenced by geopolitical situations. Besides these economic considerations, it is worth mentioning that ammonia industries in Spain are subject to the European Union Emissions Trading System (EU ETS).^35^ The EU ETS is the EU primary policy to reduce greenhouse gas emissions in which specific industries that are highly intensive in carbon buy or receive emission allowances for their direct greenhouse gas emissions.^35^ The emission allowances can be either allocated for free or purchased based on a carbon market price.^35^ In this regard, a sensitivity analysis was conducted to evaluate the potential impact of the carbon market price on green ammonia implementation projects in Spain considering a scenario in which all of the emission allowances need to be purchased.

**Figure 4:**
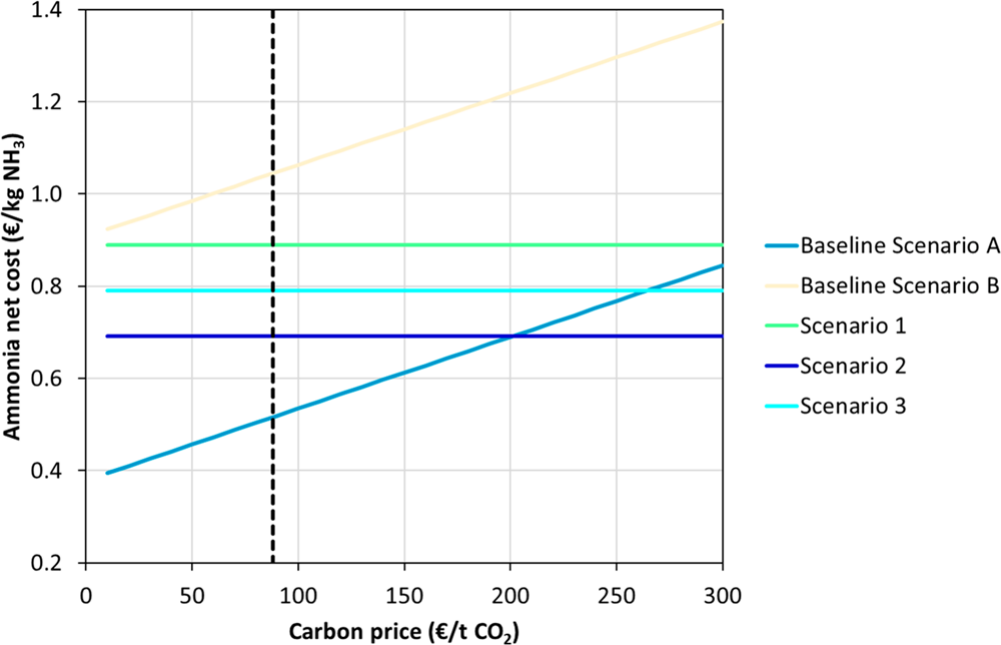
Ammonia net cost variation when the carbon price ranged between 0 and 300 € t^–1^ CO_2_ for the two gray ammonia scenarios (Baseline Scenario A, natural gas cost of 28.8 €/MWh; and Baseline Scenario B, natural gas cost of 88.9 €/MWh) and the three green ammonia scenarios (Scenario 1, 100% wind energy; Scenario 2, 100% solar energy; and Scenario 3, 50% solar and 50% wind energy). The dashed line represents the current carbon market price (7/20/2023).

Figure 4 shows the variation of the net ammonia production cost when the carbon prices ranged between 0 and 300 €/t CO_2_.^13,36^ The net ammonia production cost increased from 0.4 to 0.85 €/kg of NH_3_ in the Baseline Scenario (Alternative A) as the carbon price increased from 0 to 300 €/t of CO_2_, respectively. Figure 4 also shows that the net ammonia production costs of Scenarios 2 and 3 became lower than the Baseline Scenario (Alternative A) at carbon prices of 200 and 260 €/t of CO_2_, respectively. However, it seems unlikely that these carbon prices could be achieved, particularly considering that the current carbon price is around 90 €/t CO_2_ (dashed line) and that the maximum historical values reached 100 €/t CO_2_.^37^ Overall, these results suggest that high carbon prices could incentivize the transformation of already existing gray ammonia production plants to green ammonia production plants. However, the existing infrastructure for conventional ammonia production is set for the SMR process, which implies that changing the paradigm to green ammonia production would require (i) installing the electrolyzer, (ii) adapting the existing SMR and HB infrastructure, (iii) electrifying the whole production process, and (iv) developing the renewable energy infrastructure. For this reason, understanding the impact of retrofitting existing gray to green ammonia production plants is important for widespread implementation of these systems (out of the scope of the present manuscript).

### Opportunities and Challenges of Green Ammonia Implementation in Spain

Ammonia is the second most produced chemical in the world, and its market accounts for 91–225 billion dollars per year.^11^ This market is dominated by the agricultural sector since 80% of the ammonia manufactured is used to produce nitrogen-based fertilizers.^38^ In this regard, Spain could be an attractive country for green ammonia implementation because it has a high availability of renewable energy sources and a very active agricultural sector. Spain is one of the European Countries with the highest solar irradiation on a horizontal plane (1.48–3.56 kW/m^2^)^39^ and the fifth country in the world in terms of installed wind capacity with an accumulated power of 28 GW.^40^ Overall, wind and solar (including PV and solar thermal) represented 22.1 and 11.5% of the national electricity generation in 2022,^41^ although it is expected that their contribution could increase by 2030 to 31 and 29%, respectively.^42^ This highlights that Spain has the potential to move toward the production of green ammonia.

In recent years, the application of ammonia is moving beyond the well-known agricultural sector toward the energy sector.^4,7^ Green ammonia can be used in the energy sector as a carbon-free hydrogen carrier due to its excellent properties for energy storage and transportation.^5^ Traditionally, hydrogen has been considered as one of the most promising clean energy vectors to achieve the decarbonization of the energy and industrial sectors.^15,43^ In 2021, hydrogen demand reached 94 Mt and represented 2.5% of global energy consumption.^44^ Regarding the Spanish situation, this country has an annual production of 500,000 tons of hydrogen of which gray hydrogen represents up to 99%.^45^ However, despite most of the hydrogen being produced through traditional gray routes, Spain has great potential to move toward the production of green hydrogen. The solar PV generation in Spain is expected to increase to 40 GW by 2030, which highlights the importance to have flexible backups during the day to reduce energy curtailment.^42^ Green hydrogen application can be an interesting alternative to be used as a storage method for the non-dispatchable renewable energy generated.^46^ In this regard, Spain has emerged as one of the leading European Countries in terms of projects promoting green hydrogen production.^47^ However, using hydrogen as an energy vector faces some challenges since hydrogen features a low volumetric energy density (4.5 MJ/L for compressed H_2_) and a low boiling point (−253 °C).^5^ For this reason, ammonia represents an interesting alternative hydrogen carrier to overcome the challenges associated with hydrogen transportation and storage because this compound features a higher volumetric energy density (12.9–14.4 MJ/L) and a higher boiling point (−33 °C) than hydrogen. These features make ammonia transportation a more cost-effective option when compared with compressed or liquefied hydrogen transportation.^11^ It is worth mentioning that the Fertiberia and Iberdrola companies commissioned in Spain the largest European plant to produce green hydrogen for subsequent green ammonia production.^48^

**Figure 5:**
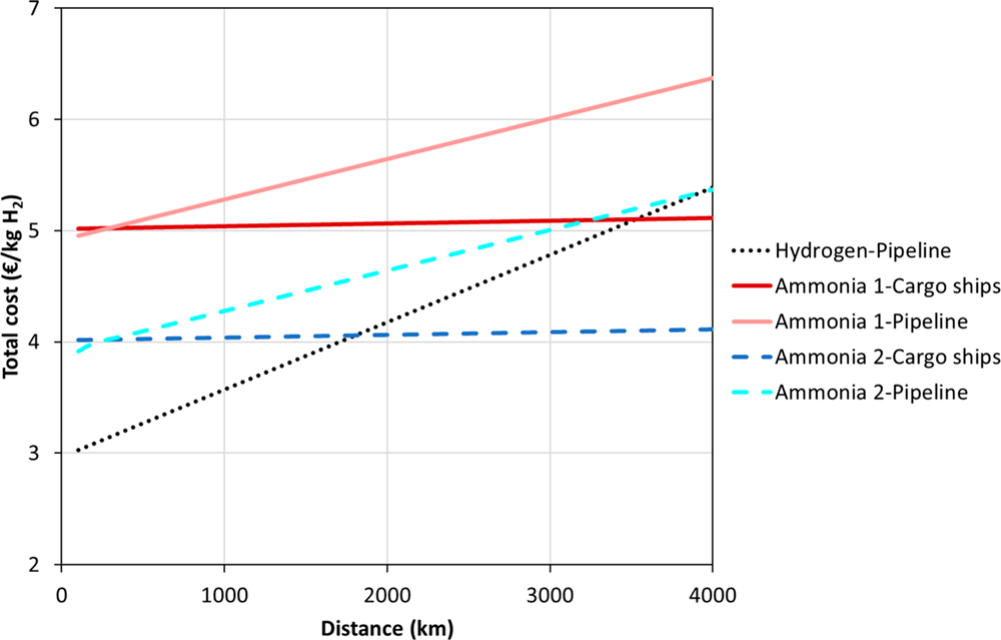
Total cost of green hydrogen and green ammonia projects considering different delivery methods for distances ranging between 0 and 4,000 km. The total cost includes the costs of production and transportation of these green energy vectors. Hydrogen-Pipeline, green H_2_ production and subsequent transportation through pipelines; Ammonia-Cargo ships, green NH_3_ production and subsequent transportation through cargo ships; Ammonia-Pipeline, green NH_3_ production and subsequent distribution through pipelines. In Alternative 1, the cracking cost of ammonia is included, while in alternative 2, the cracking cost of ammonia is not included. The transportation costs have been obtained from the literature.^11,50^

However, the use of ammonia as an energy vector deserves special attention. The use of ammonia as an energy vector reduces the transportation costs, but it implies extra conversion steps when compared with direct use of hydrogen. Figure 5 shows the impact of transport distance and delivery method on the economic prospect of using green ammonia as an energy vector when compared to green hydrogen (Figure 5). The analysis included five potential alternatives (one for green hydrogen and four for green ammonia) considering transportation distances from Spain to other European countries in the range of 100–4,000 km. The analysis included the costs concerning the conversion processes needed to produce the energy vectors (green hydrogen and ammonia), but it did not include the costs concerning the final use of the energy vector. For green ammonia, two alternatives were included: (i) Alternative 1, in which the green ammonia is cracked into hydrogen before energy production; and (ii) Alternative 2, in which the green ammonia is not cracked before energy production. Table S4 summarizes the five alternatives included. Figure 5 shows that using green hydrogen as an energy vector (distributed through pipelines) is the best alternative for relatively low distances. The results also illustrate that Alternative 1 was the less economically favorable alternative. However, Alternative 2 featured lower costs than the green hydrogen alternative for transportation distances above 1,800 km (cargo ships) and 4,000 km (pipelines), respectively. These results highlight the importance of developing efficient ammonia-fueled systems to avoid the conversion of ammonia to hydrogen before energy production and to overcome the current limitations of using ammonia as an energy vector.^38^ However, the development of ammonia-fueled systems (i.e., engines, gas turbines, or fuel cells) is still at an early stage (TRL 3–5), and further research is necessary to make this option possible from technical, environmental, and economic points of view.^11,49^

Water availability is an important factor for green ammonia and hydrogen production in Spain. According to the stoichiometry of the water electrolysis process, a minimum of 9 kg of deionized water per each kg of hydrogen produced is needed.^51^ The high demand of water needed for electrolysis contrasts with the current water scarcity since Spain is among the countries facing the most significant water scarcity conditions within Europe.^52^ Green hydrogen production by using regenerated water can be important to promote green ammonia implementation in countries facing water scarcity conditions. However, the application of regenerated water for green hydrogen production is challenging because cost-intensive technologies would be needed to achieve the deionized water quality required for electrolysis.^53^ The development of cost-effective technologies able to achieve the high-quality water needed for the electrolysis stage is important for the successful implementation of green ammonia projects in Spain.

## Conclusions

The results of this study revealed that green ammonia implementation reduced the environmental impacts related to climate change, air quality, and fossil resource scarcity when compared with gray ammonia production. Conversely, green ammonia implementation increased the environmental impacts in the land use, mineral resource scarcity, freshwater eutrophication, and terrestrial acidification categories. The techno-economic evaluation showed that gray ammonia production was the most economically favorable option under scenarios with relatively low costs of natural gas. However, green ammonia could become a less costly option when the natural gas cost and carbon prices increased. Finally, the results illustrated that green hydrogen could be a more attractive energy vector than green ammonia for relatively low transportation distances and when considering the entire supply chain (production/transportation). Nevertheless, developing efficient ammonia-fueled systems is important to make green ammonia a relevant energy vector for future decarbonization scenarios.

## Supporting Information Available

Inventory data for the different scenarios, background processes from the Ecoinvent v3.7 database, average economic parameters used for the techno-economic evaluation, summary of the five alternatives evaluated in Figure 5, schematic representation of gray ammonia production plant and green ammonia production plant, and sensitivity analysis results (PDF)

## References

[ref1] European Commission. European Green Deal. https://commission.europa.eu/strategy-and-policy/priorities-2019-2024/european-green-deal_en (accessed 2023–06–27).

[ref2] Ye D.; Tsang S. C. E. Prospects and Challenges of Green Ammonia Synthesis. Nat. Synth. 2023, 2, 612–623. 10.1038/s44160-023-00321-7.

[ref3] Ghavam S.; Vahdati M.; Wilson I. A. G.; Styring P. Sustainable Ammonia Production Processes. Frontiers in Energy Research 2021, 9, 34 10.3389/fenrg.2021.580808.

[ref4] Smith C.; Hill A. K.; Torrente-Murciano L. Current and Future Role of Haber–Bosch Ammonia in a Carbon-Free Energy Landscape. Energy Environ. Sci. 2020, 13 (2), 331–344. 10.1039/C9EE02873K.

[ref5] Lee B.; Winter L. R.; Lee H.; Lim D.; Lim H.; Elimelech M. Pathways to a Green Ammonia Future. ACS Energy Lett. 2022, 7 (9), 3032–3038. 10.1021/acsenergylett.2c01615.

[ref6] Liu X.; Elgowainy A.; Wang M. Life Cycle Energy Use and Greenhouse Gas Emissions of Ammonia Production from Renewable Resources and Industrial By-Products. Green Chem. 2020, 22 (17), 5751–5761. 10.1039/D0GC02301A.

[ref7] Salmon N.; Bañares-Alcántara R. Green Ammonia as a Spatial Energy Vector: A Review. Sustainable Energy Fuels 2021, 5 (11), 2814–2839. 10.1039/D1SE00345C.

[ref8] Nayak-Luke R.; Bañares-Alcántara R.; Wilkinson I. Green” Ammonia: Impact of Renewable Energy Intermittency on Plant Sizing and Levelized Cost of Ammonia. Ind. Eng. Chem. Res. 2018, 57 (43), 14607–14616. 10.1021/acs.iecr.8b02447.

[ref9] Rouwenhorst K. H. R.; Van der Ham A. G. J.; Mul G.; Kersten S. R. A. Islanded Ammonia Power Systems: Technology Review & Conceptual Process Design. Renewable and Sustainable Energy Reviews 2019, 114, 109339 10.1016/j.rser.2019.109339.

[ref10] Bicer Y.; Dincer I.; Zamfirescu C.; Vezina G.; Raso F. Comparative Life Cycle Assessment of Various Ammonia Production Methods. Journal of Cleaner Production 2016, 135, 1379–1395. 10.1016/j.jclepro.2016.07.023.

[ref11] Cardoso J. S.; Silva V.; Rocha R. C.; Hall M. J.; Costa M.; Eusébio D. Ammonia as an Energy Vector: Current and Future Prospects for Low-Carbon Fuel Applications in Internal Combustion Engines. Journal of Cleaner Production 2021, 296, 126562 10.1016/j.jclepro.2021.126562.

[ref12] Zhang H.; Wang L.; Van herle J.; Maréchal F.; Desideri U. Techno-Economic Comparison of Green Ammonia Production Processes. Applied Energy 2020, 259, 114135 10.1016/j.apenergy.2019.114135.

[ref13] Egerer J.; Grimm V.; Niazmand K.; Runge P. The Economics of Global Green Ammonia Trade – “Shipping Australian Wind and Sunshine to Germany. Applied Energy 2023, 334, 120662 10.1016/j.apenergy.2023.120662.

[ref14] Campion N.; Nami H.; Swisher P. R.; Vang Hendriksen P.; Münster M. Techno-Economic Assessment of Green Ammonia Production with Different Wind and Solar Potentials. Renewable and Sustainable Energy Reviews 2023, 173, 113057 10.1016/j.rser.2022.113057.

[ref15] Hermesmann M.; Müller T. E. Green, Turquoise, Blue, or Grey? Environmentally Friendly Hydrogen Production in Transforming Energy Systems. Prog. Energy Combust. Sci. 2022, 90, 100996 10.1016/j.pecs.2022.100996.

[ref16] Nosherwani S. A.; Neto R. C. Techno-Economic Assessment of Commercial Ammonia Synthesis Methods in Coastal Areas of Germany. Journal of Energy Storage 2021, 34, 102201 10.1016/j.est.2020.102201.

[ref17] Yapicioglu A.; Dincer I. A Review on Clean Ammonia as a Potential Fuel for Power Generators. Renewable and Sustainable Energy Reviews 2019, 103, 96–108. 10.1016/j.rser.2018.12.023.

[ref18] Eurostat. Natural gas price statistics. https://ec.europa.eu/eurostat/statistics-explained/index.php?title=Natural_gas_price_statistics (accessed 2023–06–27).

[ref19] Hermesmann M.; Grübel K.; Scherotzki L.; Müller T. E. Promising Pathways: The Geographic and Energetic Potential of Power-to-x Technologies Based on Regeneratively Obtained Hydrogen. Renewable and Sustainable Energy Reviews 2021, 138, 110644 10.1016/j.rser.2020.110644.

[ref20] Rakousky C.; Reimer U.; Wippermann K.; Kuhri S.; Carmo M.; Lueke W.; Stolten D. Polymer Electrolyte Membrane Water Electrolysis: Restraining Degradation in the Presence of Fluctuating Power. J. Power Sources 2017, 342, 38–47. 10.1016/j.jpowsour.2016.11.118.

[ref21] Auguadra M.; Ribó-Pérez D.; Gómez-Navarro T. Planning the Deployment of Energy Storage Systems to Integrate High Shares of Renewables: The Spain Case Study. Energy 2023, 264, 126275 10.1016/j.energy.2022.126275.

[ref22] ISO. ISO 14040:2006—Environmental Management - Life Cycle Assessment - Principles and Framework. https://www.iso.org/standard/37456.html (accessed 2022–12–15).

[ref23] ISO. ISO 14044:2006—Environmental Management - Life Cycle Assessment - Requirements and Guidelines. https://www.iso.org/standard/38498.html (accessed 2022–12–15).

[ref24] Lee K.; Liu X.; Vyawahare P.; Sun P.; Elgowainy A.; Wang M. Techno-Economic Performances and Life Cycle Greenhouse Gas Emissions of Various Ammonia Production Pathways Including Conventional, Carbon-Capturing, Nuclear-Powered, and Renewable Production. Green Chem. 2022, 24 (12), 4830–4844. 10.1039/D2GC00843B.

[ref25] Vinardell S.; Cortina J. L.; Valderrama C. Environmental and Economic Evaluation of Implementing Membrane Technologies and Struvite Crystallisation to Recover Nutrients from Anaerobic Digestion Supernatant. Bioresour. Technol. 2023, 384, 129326 10.1016/j.biortech.2023.129326. 37315623

[ref26] Valero A.; Valero A.; Calvo G.; Ortego A. Material Bottlenecks in the Future Development of Green Technologies. Renewable and Sustainable Energy Reviews 2018, 93, 178–200. 10.1016/j.rser.2018.05.041.

[ref27] Valero A.; Valero A.; Calvo G.; Ortego A.; Ascaso S.; Palacios J.-L. Global Material Requirements for the Energy Transition. An Exergy Flow Analysis of Decarbonisation Pathways. Energy 2018, 159, 1175–1184. 10.1016/j.energy.2018.06.149.

[ref28] Wang J.; Shahbaz M.; Dong K.; Dong X. Renewable Energy Transition in Global Carbon Mitigation: Does the Use of Metallic Minerals Matter?. Renewable and Sustainable Energy Reviews 2023, 181, 113320 10.1016/j.rser.2023.113320.

[ref29] Nonhebel S. Renewable Energy and Food Supply: Will There Be Enough Land?. Renewable and Sustainable Energy Reviews 2005, 9 (2), 191–201. 10.1016/j.rser.2004.02.003.

[ref30] Hernandez R. R.; Easter S. B.; Murphy-Mariscal M. L.; Maestre F. T.; Tavassoli M.; Allen E. B.; Barrows C. W.; Belnap J.; Ochoa-Hueso R.; Ravi S.; Allen M. F. Environmental Impacts of Utility-Scale Solar Energy. Renewable and Sustainable Energy Reviews 2014, 29, 766–779. 10.1016/j.rser.2013.08.041.

[ref31] van de Ven D.-J.; Capellan-Peréz I.; Arto I.; Cazcarro I.; de Castro C.; Patel P.; Gonzalez-Eguino M. The Potential Land Requirements and Related Land Use Change Emissions of Solar Energy. Sci. Rep 2021, 11 (1), 2907 10.1038/s41598-021-82042-5. 33536519 PMC7859221

[ref32] Asdrubali F.; Baldinelli G.; D’Alessandro F.; Scrucca F. Life Cycle Assessment of Electricity Production from Renewable Energies: Review and Results Harmonization. Renewable and Sustainable Energy Reviews 2015, 42, 1113–1122. 10.1016/j.rser.2014.10.082.

[ref33] Evans A.; Strezov V.; Evans T. J. Assessment of Sustainability Indicators for Renewable Energy Technologies. Renewable and Sustainable Energy Reviews 2009, 13 (5), 1082–1088. 10.1016/j.rser.2008.03.008.

[ref34] Su C. W.; Qin M.; Chang H.-L.; Ţăran A.-M. Which Risks Drive European Natural Gas Bubbles? Novel Evidence from Geopolitics and Climate. Resources Policy 2023, 81, 103381 10.1016/j.resourpol.2023.103381.

[ref35] European Parliament. Directive 2003/87/EC of the European Parliament and of the Council of 13 October 2003 Establishing a System for Greenhouse Gas Emission Allowance Trading within the Union and Amending Council Directive 96/61/EC (Text with EEA Relevance); 2018. http://data.europa.eu/eli/dir/2003/87/2018-04-08/eng (accessed 2023–06–27).

[ref36] Rennert K.; Errickson F.; Prest B. C.; Rennels L.; Newell R. G.; Pizer W.; Kingdon C.; Wingenroth J.; Cooke R.; Parthum B.; Smith D.; Cromar K.; Diaz D.; Moore F. C.; Müller U. K.; Plevin R. J.; Raftery A. E.; Ševčíková H.; Sheets H.; Stock J. H.; Tan T.; Watson M.; Wong T. E.; Anthoff D. Comprehensive Evidence Implies a Higher Social Cost of CO2. Nature 2022, 610 (7933), 687–692. 10.1038/s41586-022-05224-9. 36049503 PMC9605864

[ref37] EMBER. Carbon Price Tracker. https://ember-climate.org/data/data-tools/carbon-price-viewer/ (accessed 2023–06–27).

[ref38] Chatterjee S.; Parsapur R. K.; Huang K.-W. Limitations of Ammonia as a Hydrogen Energy Carrier for the Transportation Sector. ACS Energy Lett. 2021, 6 (12), 4390–4394. 10.1021/acsenergylett.1c02189.

[ref39] Heras-Saizarbitoria I.; Cilleruelo E.; Zamanillo I. Public Acceptance of Renewables and the Media: An Analysis of the Spanish PV Solar Experience. Renewable and Sustainable Energy Reviews 2011, 15 (9), 4685–4696. 10.1016/j.rser.2011.07.083.

[ref40] Spanish Wind Energy Association. Wind energy in Spain. https://aeeolica.org/sobre-la-eolica/la-eolica-en-espana/ (accessed 2023–04–26).

[ref41] Red Eléctrica. REData - Estructura generacion. https://www.ree.es/es/datos/generacion/estructura-generacion (accessed 2023–04–26).

[ref42] Gómez-Calvet R.; Martínez-Duart J. M.; Gómez-Calvet A. R. The 2030 Power Sector Transition in Spain: Too Little Storage for so Many Planned Solar Photovoltaics?. Renewable and Sustainable Energy Reviews 2023, 174, 113094 10.1016/j.rser.2022.113094.

[ref43] Ball M.; Wietschel M. The Future of Hydrogen – Opportunities and Challenges. Int. J. Hydrogen Energy 2009, 34 (2), 615–627. 10.1016/j.ijhydene.2008.11.014.

[ref44] International Energy Agency. Global Hydrogen Review 2022—Analysis. https://www.iea.org/reports/global-hydrogen-review-2022 (accessed 2023–04–26).

[ref45] Ministerio para la Transición Ecológica. Hoja de Ruta Del Hidrógeno; 2020. https://www.miteco.gob.es/es/ministerio/planes-estrategias/hidrogeno/default.aspx (accessed 2023–07–18).

[ref46] Capurso T.; Stefanizzi M.; Torresi M.; Camporeale S. M. Perspective of the Role of Hydrogen in the 21st Century Energy Transition. Energy Conversion and Management 2022, 251, 114898 10.1016/j.enconman.2021.114898.

[ref47] Matute G.; Yusta J. M.; Naval N. Techno-Economic Model and Feasibility Assessment of Green Hydrogen Projects Based on Electrolysis Supplied by Photovoltaic PPAs. Int. J. Hydrogen Energy 2023, 48 (13), 5053–5068. 10.1016/j.ijhydene.2022.11.035.

[ref48] Iberdrola. Iberdrola commissions the largest green hydrogen plant for industrial use in Europe. https://www.iberdrola.com/about-us/what-we-do/green-hydrogen/puertollano-green-hydrogen-plant (accessed 2023–04–26).

[ref49] Herbinet O.; Bartocci P.; Grinberg Dana A. On the Use of Ammonia as a Fuel – A Perspective. Fuel Communications 2022, 11, 100064 10.1016/j.jfueco.2022.100064.

[ref50] International Energy Agency. The Future of Hydrogen. https://www.iea.org/reports/the-future-of-hydrogen (accessed 2023–07–18).

[ref51] Beswick R. R.; Oliveira A. M.; Yan Y. Does the Green Hydrogen Economy Have a Water Problem?. ACS Energy Lett. 2021, 6 (9), 3167–3169. 10.1021/acsenergylett.1c01375.

[ref52] Molina V. G.; Casañas A. Reverse Osmosis, a Key Technology in Combating Water Scarcity in Spain. Desalination 2010, 250 (3), 950–955. 10.1016/j.desal.2009.09.079.

[ref53] Simoes S. G.; Catarino J.; Picado A.; Lopes T. F.; di Berardino S.; Amorim F.; Gírio F.; Rangel C. M.; Ponce de Leão T. Water Availability and Water Usage Solutions for Electrolysis in Hydrogen Production. Journal of Cleaner Production 2021, 315, 128124 10.1016/j.jclepro.2021.128124.

