# Supplementary material for: Sustainability Assessment of Green Ammonia Production To Promote Industrial Decarbonization in Spain
Source: ACS Sustain Chem Eng. 2023 Oct 25;11(44):15975–83. doi: 10.1021/acssuschemeng.3c04694 (PMC10631447; doi:10.1021/acssuschemeng.3c04694)
Supplement: Supplementary file 1 — sc3c04694_si_001.pdf [file sc3c04694_si_001.pdf]

SUPPORTING INFORMATION

Sustainability assessment of green ammonia production to promote industrial decarbonization in Spain

Sergi Vinardell [*,†,‡], Palina Nicolas[†,‡], Ana María Sastre[†,‡], Jose Luis Cortina[†,‡,§], César Valderrama[†,‡]

[†]Chemical Engineering Department, Escola d'Enginyeria de Barcelona Est (EEBE), Universitat Politècnica de Catalunya (UPC)-BarcelonaTECH, C/ Eduard Maristany 10-14, Campus Diagonal-Besòs, 08930 Barcelona, Spain

[‡]Barcelona Research Center for Multiscale Science and Engineering, Campus Diagonal-Besòs, 08930 Barcelona, Spain.

[§]CETaqua, Carretera d'Esplugues, 75, 08940 Cornellà de Llobregat, Spain.

*Corresponding author (sergi.vinardell@upc.edu)

Number of pages: 10

Number of tables: 4

Number of figures: 2

Table S1: Inventory data for the four scenarios under study. The data are referred to the functional unit (1 kg of NH_3 produced).

				Baseline Scenario	Scenario 1	Scenario 2	Scenario 3
Inputs	Steam methane reforming	Concrete[a]	m^3	8.4×10^{-7}	-	-	-
		Steel[a]	kg	6.4×10^{-4}	-	-	-
		Aluminium[a]	kg	5.3×10^{-6}	-	-	-
		Iron[a]	kg	7.9×10^{-6}	-	-	-
		Natural gas[b]	Nm^3	8.6×10^{-1}	-	-	-
		Cooling water[b]	m^3	7.1×10^{-2}	-	-	-
		Process water[b]	kg	1.2×10^{0}	-	-	-
	Haber Bosch	Concrete[c]	m^3	3.1×10^{-7}	3.1×10^{-7}	3.1×10^{-7}	3.1×10^{-7}
		Steel[c]	kg	1.1×10^{-3}	1.1×10^{-3}	1.1×10^{-3}	1.1×10^{-3}
		High alloyed steel[c]	kg	6.6×10^{-4}	6.6×10^{-4}	6.6×10^{-4}	6.6×10^{-4}
		Electronics[c]	kg	1.1×10^{-4}	1.1×10^{-4}	1.1×10^{-4}	1.1×10^{-4}
		Fe-catalyst[d]	kg	1.1×10^{-5}	1.1×10^{-5}	1.1×10^{-5}	1.1×10^{-5}
		Electricity[e]	kWh	-	3.6×10^{0}	3.6×10^{0}	3.6×10^{0}
	Electrolysis	Concrete[f]	m^3	-	1.4×10^{-7}	1.4×10^{-7}	1.4×10^{-7}
		High alloyed steel[f]	kg	-	1.3×10^{-4}	1.3×10^{-4}	1.3×10^{-4}
		Low alloyed steel[f]	kg	-	2.9×10^{-4}	2.9×10^{-4}	2.9×10^{-4}
		Titanium[f]	kg	-	6.4×10^{-5}	6.4×10^{-5}	6.4×10^{-5}
		Cooper[f]	kg	-	6.6×10^{-6}	6.6×10^{-6}	6.6×10^{-6}
		Tetrafluoroethylene[f]	kg	-	1.9×10^{-6}	1.9×10^{-6}	1.9×10^{-6}
		Activated carbon[f]	kg	-	1.1×10^{-6}	1.1×10^{-6}	1.1×10^{-6}
		Platinum[f]	kg	-	9.2×10^{-9}	9.2×10^{-9}	9.2×10^{-9}
		Aluminium[f]	kg	-	9.3×10^{-6}	9.3×10^{-6}	9.3×10^{-6}
		Iridium[f]	kg	-	9.1×10^{-8}	9.1×10^{-8}	9.1×10^{-8}
		Deionized water[b]	kg	-	2.1×10^{0}	2.1×10^{0}	2.1×10^{0}

		Electricity[b]	kWh	-	9.7×10^{0}	9.7×10^{0}	9.7×10^{0}
	Cryogenic distillation	Air separation facility[g]	items	-	3.6×10^{-10}	3.6×10^{-10}	3.6×10^{-10}
		Cooling water[g]	m^3	-	1.8×10^{-2}	1.8×10^{-2}	1.8×10^{-2}
		Electricity[h]	kWh	-	9.1×10^{-2}	9.1×10^{-2}	9.1×10^{-2}
	Hydrogen storage	Electricity for H_2 compression[i]	kWh	-	1.7×10^{-1}	1.7×10^{-1}	1.7×10^{-1}
	Li-ion battery	Li-ion battery[j]	kg	-	2.1×10^{-4}	2.4×10^{-4}	2.3×10^{-4}
Outputs	Emissions to air	Carbon dioxide[b]	kg	1.5×10^{0}	-	-	-
		Oxygen[b]	kg	5.0×10^{-2}	-	-	-
		Nitrogen[b]	kg	2.9×10^{0}	-	-	-
		Water[b]	kg	5.1×10^{-1}	-	-	-
	Products and by-products	Ammonia	kg	1.0×10^{0}	1.0×10^{0}	1.0×10^{0}	1.0×10^{0}
		Oxygen[k]	kg	-	1.7×10^{0}	1.7×10^{0}	1.7×10^{0}

[a]Calculated from data provided by Spath and Mann[1].

[b]Calculated from Hermesmann and Müller[2] data.

[c]Calculated considering the contribution of the different parts from a chemical facility from Althaus et al.[3] data.

[d]Calculated considering the weight hourly space velocity of 60,000 mL/g/h reported by Wang et al.[4].

[e]Obtained from Rouwenhorst et al.[5].

[f]Calculated from Hermesmann and Müller[2] and Bareiß et al.[6] data.

[g]Obtained from Ecoinvent v3.7 database.

[h]Obtained from Rouwenhorst et al.[5].

[i]Obtained from Campion et al.[7].

[j]Calculated considering a battery energy density of 275 Wh/kg battery[8] and the annual operating hours of wind onshore (2300 h) and PV (1800 h) systems in Spain[9].

[k]Calculated from the oxygen produced in the electrolyser and air cryogenic distillation processes.

Table S2: Background processes from the Ecoinvent v3.7 database.

	**Ecoinvent process**
Concrete	market for concrete, normal | concrete, normal | Cutoff, U - RoW
Steel	market for steel, unalloyed | steel, unalloyed | Cutoff, U - GLO
High alloyed steel	market for steel, chromium steel 18/8 | steel, chromium steel 18/8 | Cutoff, U - GLO
Low alloyed steel	market for steel, low-alloyed | steel, low-alloyed | Cutoff, U - GLO
Aluminium	market for aluminium, cast alloy | aluminium, cast alloy | Cutoff, U - GLO
Cast Iron	market for cast iron | cast iron | Cutoff, U - GLO
Electronics	market for electronics, for control units | electronics, for control units | Cutoff, U - GLO
Titanium	market for titanium, primary | titanium, primary | Cutoff, U - GLO
Cooper	market for copper, cathode | copper, cathode | Cutoff, U - GLO
Tetrafluoroethylene	market for tetrafluoroethylene | tetrafluoroethylene | Cutoff, U - GLO
Activated carbon	market for activated carbon, granular | activated carbon, granular | Cutoff, U - GLO
Platinum	market for platinum | platinum | Cutoff, U - GLO
Air separation facility	air separation facility construction | air separation facility | Cutoff, U - RER
Li-ion battery	market for battery, Li-ion, rechargeable, prismatic | battery, Li-ion, rechargeable, prismatic | Cutoff, U - GLO
Renewable electricity from solar photovoltaic	electricity production, photovoltaic, 570kWp open ground installation, multi-Si | electricity, low voltage | Cutoff, U - ES
Electricity from wind	electricity production, wind, >3MW turbine, onshore | electricity, high voltage | Cutoff, U - ES
Natural gas	market for natural gas, high pressure | natural gas, high pressure | Cutoff, U - ES
Process water	market for tap water | tap water | Cutoff, U - Europe without Switzerland
Deionized water	market for water, deionised | water, deionised | Cutoff, U - Europe without Switzerland
Fe-catalyst	market for magnetite | magnetite | Cutoff, U - GLO

Table S3: Average economic parameters used for the techno-economic evaluation.

Parameter	Unit	Value	Reference
Steam methane reforming[a]	€/kg H_2/d	1437	[10]
Haber Bosch	€/kg NH_3/d	186	[10]
Electrolyser	€/kW	1900	[5,11]
Cryogenic distillation	€/kg N_2/d	99	[10]
Li-ion battery	€/kWh	362	[7]
Hydrogen storage	€/kg H_2	461	[7]
Wind electricity cost	€/kWh	0.055	[12]
Solar photovoltaic electricity cost	€/kWh	0.04	[12]
Fe-based catalyst	€/kg	20.9	[13]
Natural gas cost (Alternative A)	€/MWh	28.8	[14]
Natural gas cost (Alternative B)	€/MWh	88.9	[14]
Maintenance	% of CAPEX	1.5	[15]
Water	€/m^3	0.6	[16]
Oxygen revenue	€/kg O_2	0.16	[17]
Pipeline transportation hydrogen	€/t_{H2}/km	0.60	[18]
Pipeline transportation ammonia	€/t_{H2}/km	0.36	[18]
Maritime transportation ammonia	€/t_{H2}/km	0.02	[18,19]
Ammonia cracking	€/kg_{H2}	1	[20]

[a]The capital cost of the steam methane reforming (SMR) process includes the SMR reactors, water-gas shift, CO_2 scrubber and methanation stages.

Table S4: Summary of the five alternatives evaluated in Figure 5.

**Alternatives**	**Description**
Hydrogen-Pipeline	This alternative includes the total cost of producing and transporting green hydrogen through pipelines.
Ammonia 1-Cargo ships	This alternative includes the total cost of producing and transporting green ammonia through cargo ships, as well as the cost of cracking ammonia intro hydrogen before energy production.
Ammonia 1-Pipeline	This alternative includes the total cost of producing and transporting green ammonia through pipelines, as well as the cost of cracking ammonia intro hydrogen before energy production.
Ammonia 2-Cargo ships	This alternative includes the total cost of producing and transporting green ammonia through cargo ships.
Ammonia 2-Pipeline	This alternative includes the total cost of producing and transporting green ammonia through pipelines.

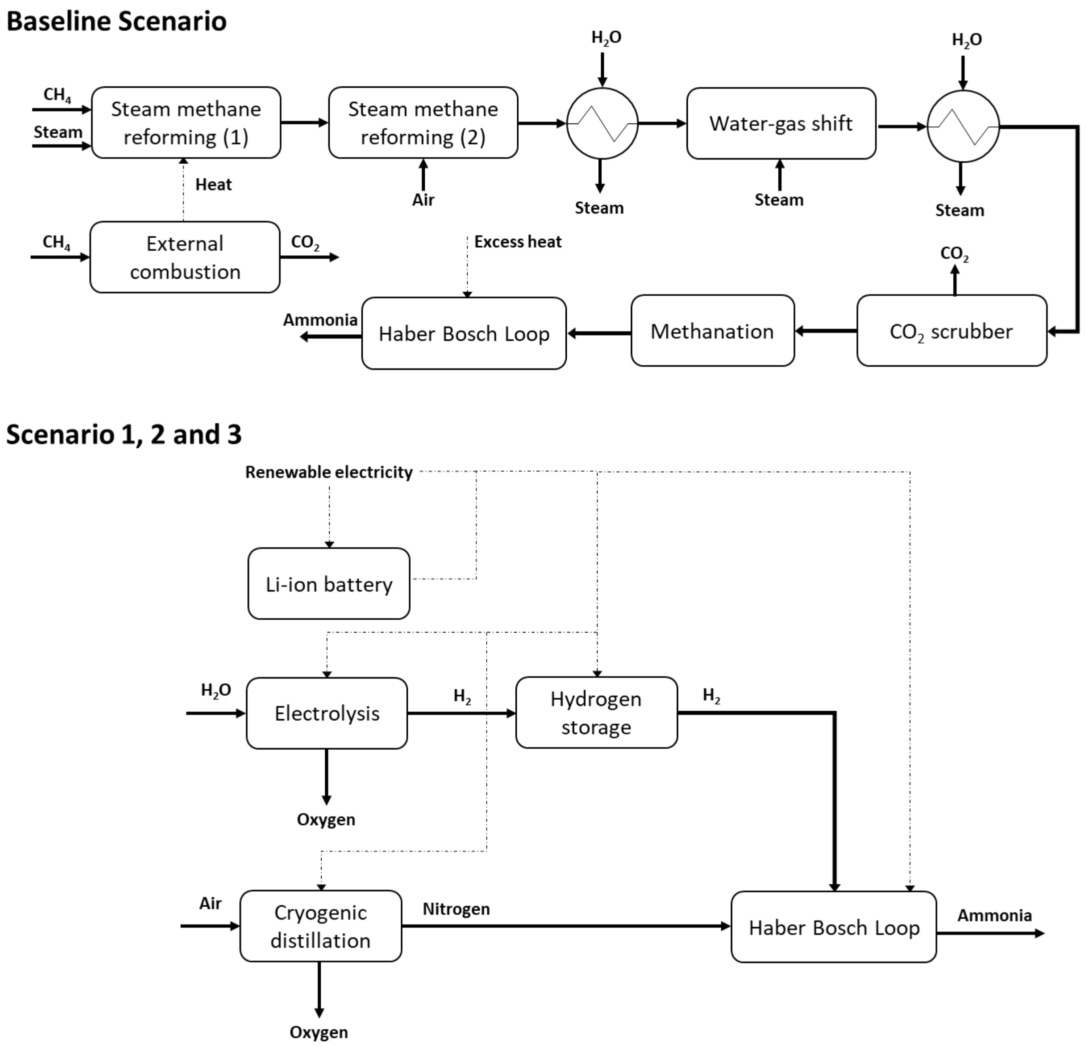

Figure S1: Schematic representation of grey ammonia production plant (top) and green ammonia production plant (bottom).

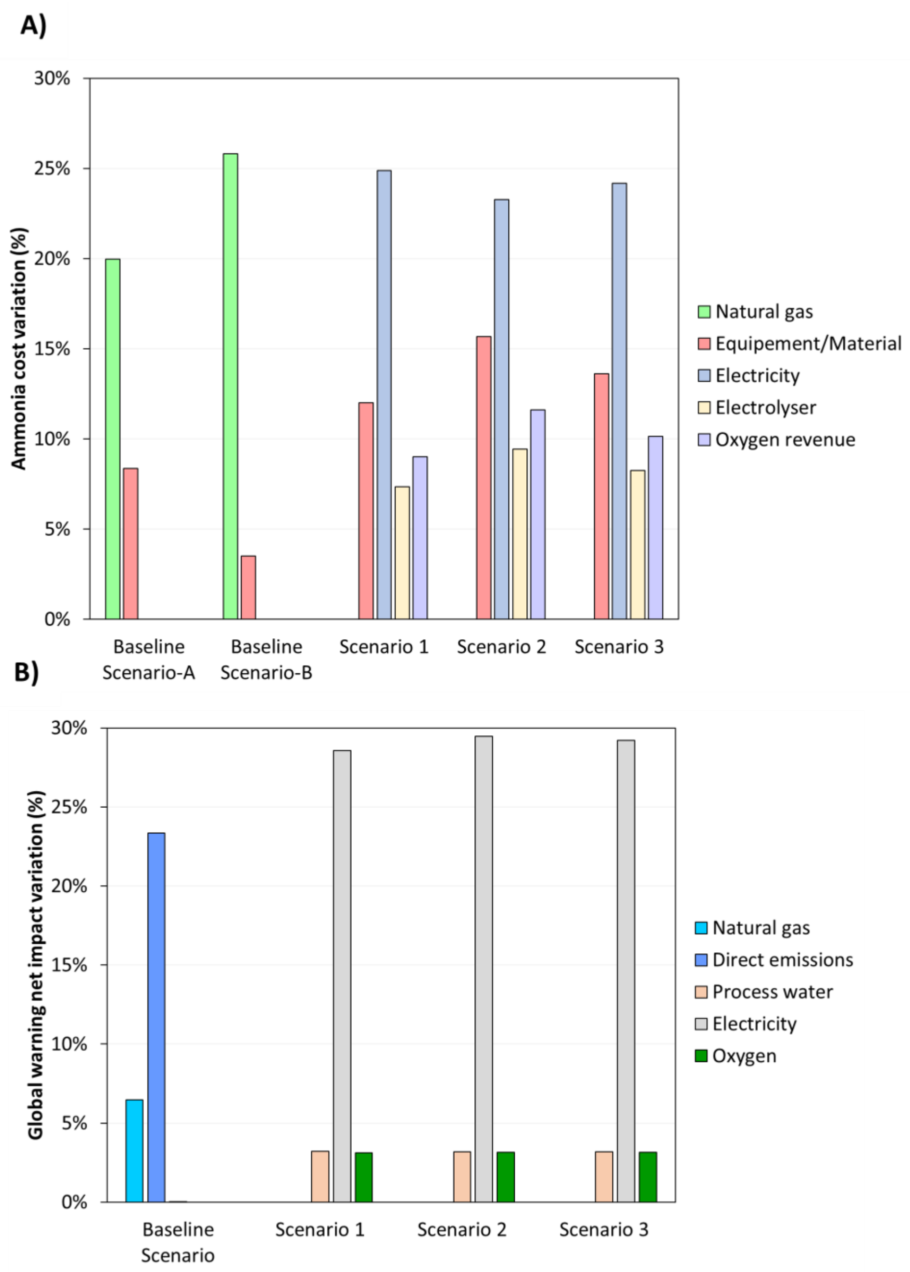

Figure S2: Sensitivity analysis for a 30% variation (in absolute terms) of the most important environmental and economic parameters for (A) ammonia cost and (B) global warming net impact.

References

(1) Spath, P. L.; Mann, M. K. Life Cycle Assessment of Hydrogen Production via Natural Gas Steam Reforming (No.NREL/TP-570-27637). **2000**. National Renewable Energy Lab. https://doi.org/10.2172/764485.

(2) Hermesmann, M.; Müller, T. E. Green, Turquoise, Blue, or Grey? Environmentally Friendly Hydrogen Production in Transforming Energy Systems. *Progress in Energy and Combustion Science* **2022**, *90*, 100996. https://doi.org/10.1016/j.pecs.2022.100996.

(3) Althaus, H. J., Chudacoff, M., Hischier, R., Jungbluth, N., Osses, M., & Primas, A. Life cycle inventories of chemicals. *Ecoinvent report* **2007**, 2.

(4) Wang, P.; Chang, F.; Gao, W.; Guo, J.; Wu, G.; He, T.; Chen, P. Breaking Scaling Relations to Achieve Low-Temperature Ammonia Synthesis through LiH-Mediated Nitrogen Transfer and Hydrogenation. *Nature Chem* **2017**, *9* (1), 64–70. https://doi.org/10.1038/nchem.2595.

(5) Rouwenhorst, K. H. R.; Van der Ham, A. G. J.; Mul, G.; Kersten, S. R. A. Islanded Ammonia Power Systems: Technology Review & Conceptual Process Design. *Renewable and Sustainable Energy Reviews* **2019**, *114*, 109339. https://doi.org/10.1016/j.rser.2019.109339.

(6) Bareiß, K.; de la Rua, C.; Möckl, M.; Hamacher, T. Life Cycle Assessment of Hydrogen from Proton Exchange Membrane Water Electrolysis in Future Energy Systems. *Applied Energy* **2019**, *237*, 862–872. https://doi.org/10.1016/j.apenergy.2019.01.001.

(7) Campion, N.; Nami, H.; Swisher, P. R.; Vang Hendriksen, P.; Münster, M. Techno-Economic Assessment of Green Ammonia Production with Different Wind and Solar Potentials. *Renewable and Sustainable Energy Reviews* **2023**, *173*, 113057. https://doi.org/10.1016/j.rser.2022.113057.

(8) Wang, C.-Y.; Liu, T.; Yang, X.-G.; Ge, S.; Stanley, N. V.; Rountree, E. S.; Leng, Y.; McCarthy, B. D. Fast Charging of Energy-Dense Lithium-Ion Batteries. *Nature* **2022**, *611* (7936), 485–490. https://doi.org/10.1038/s41586-022-05281-0.

(9) Auguadra, M.; Ribó-Pérez, D.; Gómez-Navarro, T. Planning the Deployment of Energy Storage Systems to Integrate High Shares of Renewables: The Spain Case Study. *Energy* **2023**, *264*, 126275. https://doi.org/10.1016/j.energy.2022.126275.

(10) Nosherwani, S. A.; Neto, R. C. Techno-Economic Assessment of Commercial Ammonia Synthesis Methods in Coastal Areas of Germany. *Journal of Energy Storage* **2021**, *34*, 102201. https://doi.org/10.1016/j.est.2020.102201.

(11) Hermesmann, M.; Grübel, K.; Scherotzki, L.; Müller, T. E. Promising Pathways: The Geographic and Energetic Potential of Power-to-x Technologies Based on Regeneratively Obtained Hydrogen. *Renewable and Sustainable Energy Reviews* **2021**, *138*, 110644. https://doi.org/10.1016/j.rser.2020.110644.

(12) Kost, C.; Shammugam, S.; Fluri, V.; Peper, D.; Memar, A. D.; Schlegl, T. Levelized Cost of Electricity - Renewable Energy Technologies - Fraunhofer ISE. **2021**. Fraunhofer Institute for Solar Energy Systems ISE. https://www.ise.fraunhofer.de/en/publications/studies/cost-of-electricity.html (accessed 2023-07-19).

(13) Akbari, M.; Oyedun, A. O.; Kumar, A. Ammonia Production from Black Liquor Gasification and Co-Gasification with Pulp and Waste Sludges: A Techno-Economic Assessment. *Energy* **2018**, *151*, 133–143. https://doi.org/10.1016/j.energy.2018.03.056.

(14) Eurostat. *Natural gas price statistics*. https://ec.europa.eu/eurostat/statistics-explained/index.php?title=Natural_gas_price_statistics (accessed 2023-06-27).

(15) Fúnez Guerra, C.; Reyes-Bozo, L.; Vyhmeister, E.; Jaén Caparrós, M.; Salazar, J. L.; Clemente-Jul, C. Technical-Economic Analysis for a Green Ammonia Production Plant

in Chile and Its Subsequent Transport to Japan. *Renewable Energy* **2020**, *157*, 404–414. https://doi.org/10.1016/j.renene.2020.05.041.
(16) Ochs, P.; Martin, B.; Germain-Cripps, E.; Stephenson, T.; van Loosdrecht, M.; Soares, A. Techno-Economic Analysis of Sidestream Ammonia Removal Technologies: Biological Options versus Thermal Stripping. *Environmental Science and Ecotechnology* **2023**, *13*, 100220. https://doi.org/10.1016/j.ese.2022.100220.
(17) Zhang, H.; Wang, L.; Van herle, J.; Maréchal, F.; Desideri, U. Techno-Economic Comparison of Green Ammonia Production Processes. *Applied Energy* **2020**, *259*, 114135. https://doi.org/10.1016/j.apenergy.2019.114135.
(18) International Energy Agency (IEA). *The Future of Hydrogen*. **2021**, Report. https://www.iea.org/reports/the-future-of-hydrogen (accessed 2021-07-21).
(19) Cardoso, J. S.; Silva, V.; Rocha, R. C.; Hall, M. J.; Costa, M.; Eusébio, D. Ammonia as an Energy Vector: Current and Future Prospects for Low-Carbon Fuel Applications in Internal Combustion Engines. *Journal of Cleaner Production* **2021**, *296*, 126562. https://doi.org/10.1016/j.jclepro.2021.126562.
(20) Salmon, N.; Bañares-Alcántara, R. Green Ammonia as a Spatial Energy Vector: A Review. *Sustainable Energy Fuels* **2021**, *5* (11), 2814–2839. https://doi.org/10.1039/D1SE00345C.
